# A response to Germain, et al. The effect of enzyme replacement therapy on clinical outcomes in male patients with Fabry disease: A systematic literature review by a European panel of experts. *Mol Genet Metab Rep* Feb 6 2019

**DOI:** 10.1016/j.ymgmr.2019.100471

**Published:** 2019-04-13

**Authors:** Andrey Gurevich, Jӧrn Schenk, Hartmann Wellhoefer, Vasiliki Kalampoki

**Affiliations:** Shire, A Takeda company, Zug, Switzerland

Dear editor,

*Molecular Genetics and Metabolism Reports* recently published an article by Germain and colleagues entitled, “The effect of enzyme replacement therapy on clinical outcomes in male patients with Fabry disease: A systematic literature review by a European panel of experts” [1]. In their manuscript, the authors reviewed the clinical efficacy of enzyme replacement therapy in male patients from 166 publications through January 2017. The authors aimed to summarize, cite, and extract outcomes from each publication. However, an assessment of the review against the Preferred Reporting Items for Systematic reviews and Meta-Analyses (PRISMA) guidelines indicates that several domains needed to evaluate review quality are missing from the current publication [2]. This review lacks a detailed methodology, although cited has yet to be published, of how they searched for, screened, and excluded publications. Hence, it is exceedingly difficult to assess the validity of the methodology and findings and the extent of any possible bias. At minimum, the authors should have sought to publish the methodology prior to, and not after, the publication of the literature review. In addition, we have found the following: (1) that the objective of the study was unclear from the introduction and rationale; (2) there was no risk bias assessment of individual studies on the outcomes level and no statement on how this may impact the conclusions of the analysis; (3) there was no risk bias assessment across studies; and (4) there was little to no discussion of limitations at study or review level. We would argue that in light of these discrepancies, the authors' conclusion that “consolidated evidence suggests a dose effect” for multiple outcomes, may not be supported.
